# Inherited Hairlessness: A Case Study of Familial Congenital Atrichia

**DOI:** 10.7759/cureus.37608

**Published:** 2023-04-15

**Authors:** Mounika Nagarani Tunuguntla, Bejoi Mathew, Afreen Quadri, Swetha Movva, Prithvi Raghavan, Shreyaa Rajpal, Mihirkumar P Parmar, Madhu M

**Affiliations:** 1 Internal Medicine, Guntur Medical College, Guntur, IND; 2 Internal Medicine, Sri Devaraj Urs Medical College, Kolar, IND; 3 Pediatrics, Dr. VRK Women’s Medical College, Hyderabad, IND; 4 Pediatrics, Narayana Medical College, Nellore, IND; 5 Internal Medicine, Osmania Medical College, Hyderabad, IND; 6 Internal Medicine, Gujarat Medical Education and Research Society (GMERS) Medical College, Vadnagar, IND; 7 Dermatology, SS Institute of Medical Sciences and Research Centre, Davangere, IND

**Keywords:** atrichia congenita, autosomal recessive, human hairless gene, loss of scalp hair, human hairless (hr) gene

## Abstract

An uncommon disorder known as atrichia congenita with ectodermal defects (isolated type) can present with the complete absence of hair at birth or can cause the scalp hair to fall out between the ages of one to six months, after that no new hair growth occurs. Patients don’t develop pubic and axillary hairs in addition to lacking or having scant brow, eyelash, and body hair. It could develop independently or in tandem with other problems. Isolated congenital alopecia has been reported to occur in both sporadic and familial forms. Although dominant or unevenly dominant inheritance has been found in rare families, the isolated family form often inherits in an autosomal recessive manner. In this case report, we present a rare case of familial congenital atrichia in a 16-year-old girl. There can be a genetic component to her illness because both her mother and father also show some of the clinical features.

## Introduction

Congenital atrichia is an uncommon genetic disease, and it is one of a group of disorders that presents as congenital alopecia, the others being Moynahan syndrome, progeria, hidrotic ectodermal dysplasia, vitamin D-dependent rickets type IIA, and alopecia universalis [1]. Dental aplasia has been discovered to be a common link with congenital alopecia, and abnormal sweat glands, impaired taste and smell, and epidermolysis bullosa have been identified as uncommon associations [2, 3]. It belongs to a spectrum of diseases known as ectodermal dysplasia (ED), which was originally described in the 1970s [4]. EDs, according to Freire-Maia are "congenital disorders characterised by alterations in two or more ectodermal structures, involving at least one in the hair, teeth, nails, or sweat glands" [5, 6]. Congenital atrichia is a condition in which a person is born without hair, and it is typically inherited as an autosomal recessive trait, meaning that the affected person has two copies of the defective gene, one from each parent. It can be present in sporadic or familial forms. Familial cases of congenital atrichia are even rarer, as the mutated gene is usually not present in the family history. It can be present in isolation or with associated defects. In the family, the gene locus is on chromosome 8p21-22, and the human hairless gene (HR) mutation produces the clinical picture of congenital atrichia [7].

In this case study, we describe a rare instance of family congenital atrichia in a 16-year-old girl. Zlotogorski et al. did define the initial standards for determining whether an individual has atrichia with papular lesions [8], but they were later revised by Yip et al. We have used the revised criteria proposed by Yip et al. [3]. This case study is intended to improve our understanding of the condition, its management, and the social, psychological, and other elements that influence it, making this case particularly interesting and noteworthy.

## Case presentation

**Figure 1:**
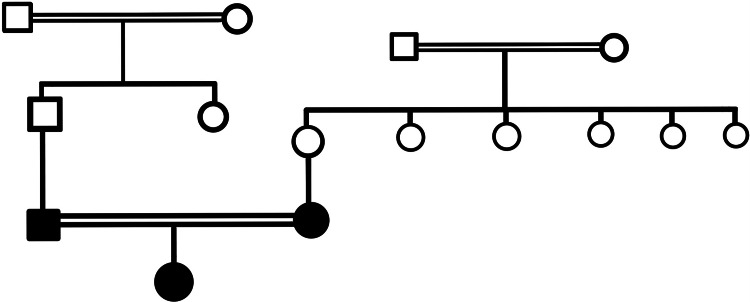
PEDIGREE Squares represent males, circles represent females, single horizontal lines connecting square and circle indicate marriage, two horizontal lines connecting square and circle indicate consanguineous marriage, half-shaded symbols indicate carrier individuals, and fully-shaded symbols indicate affected individuals.

A 16-year-old girl was discovered in a medical camp with no hair on her entire body. According to further inquiry, the patient never had any hair on her body. Moreover, she reported papular skin blemishes since infancy. The teenager was born from a third-degree consanguineous marriage and was the second-born child. Her parents were informed at the time of her birth that she had inherited the disease from her father. Her father has no any scalp hair and rest everywhere on the body he has hair. The clinical manifestation of her father and mother's family history is significant, though to differing degrees (Figure 1).

**Figure 2:**
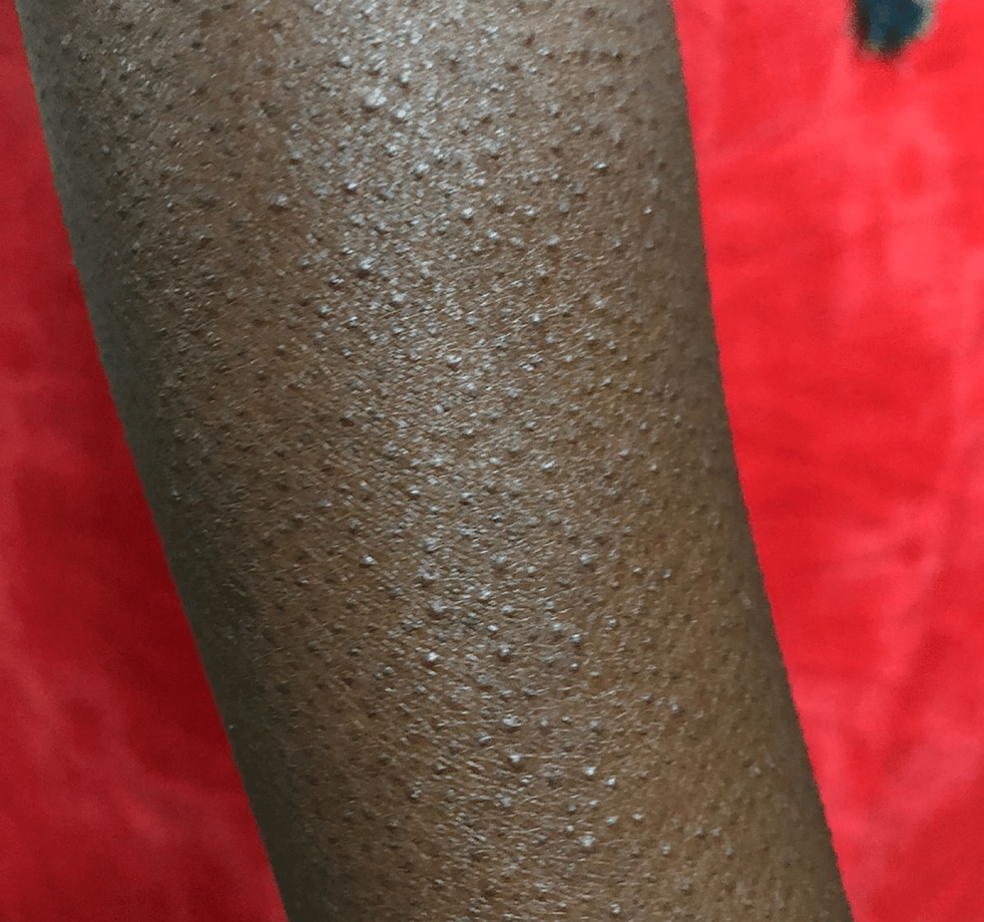
Papular skin lesions on the forearm of the girl’s mother

**Figure 3:**
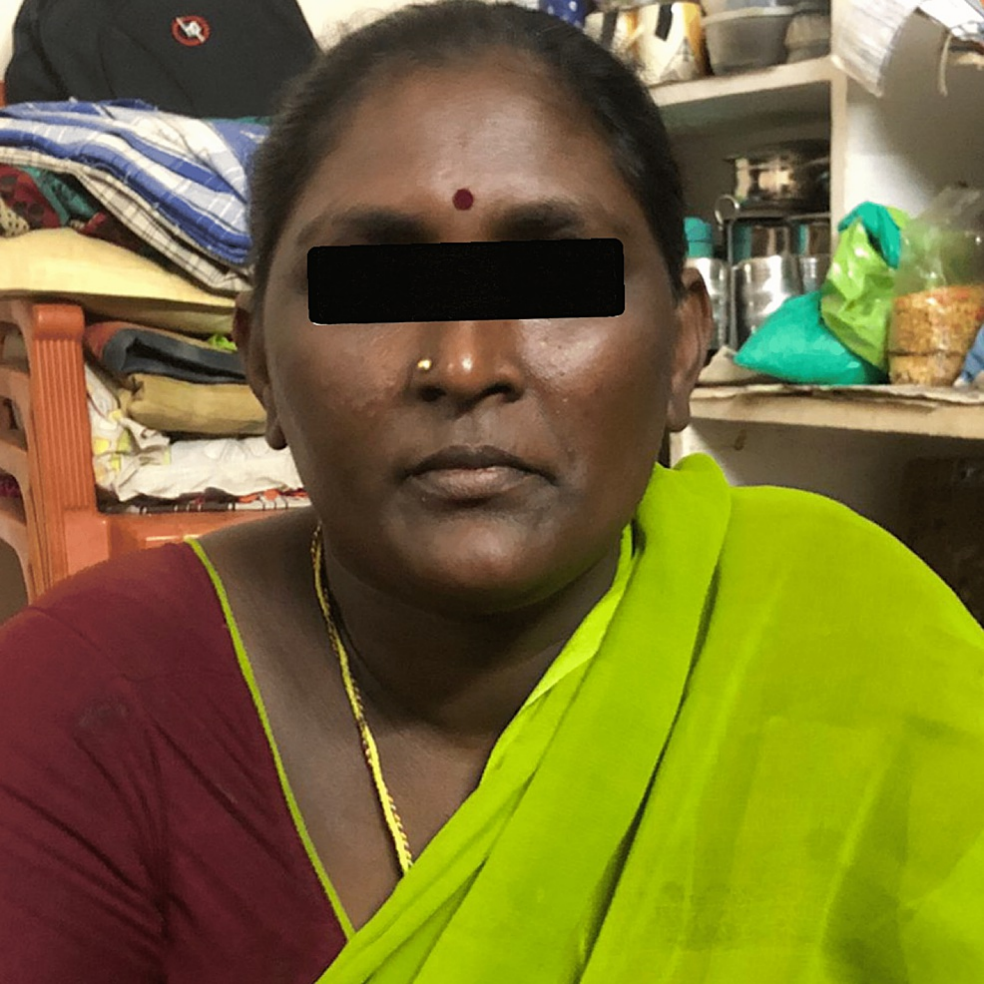
Patient's mother with normal hair, eyelashes and eyebrows

Her mother has already papular skin lesions, most prominently on the forearm, but present all over the body from birth, with normal hair all over the body, including eyebrows, eyelashes, axillary, and pubic hair (Figures 2, 3).

**Figure 4:**
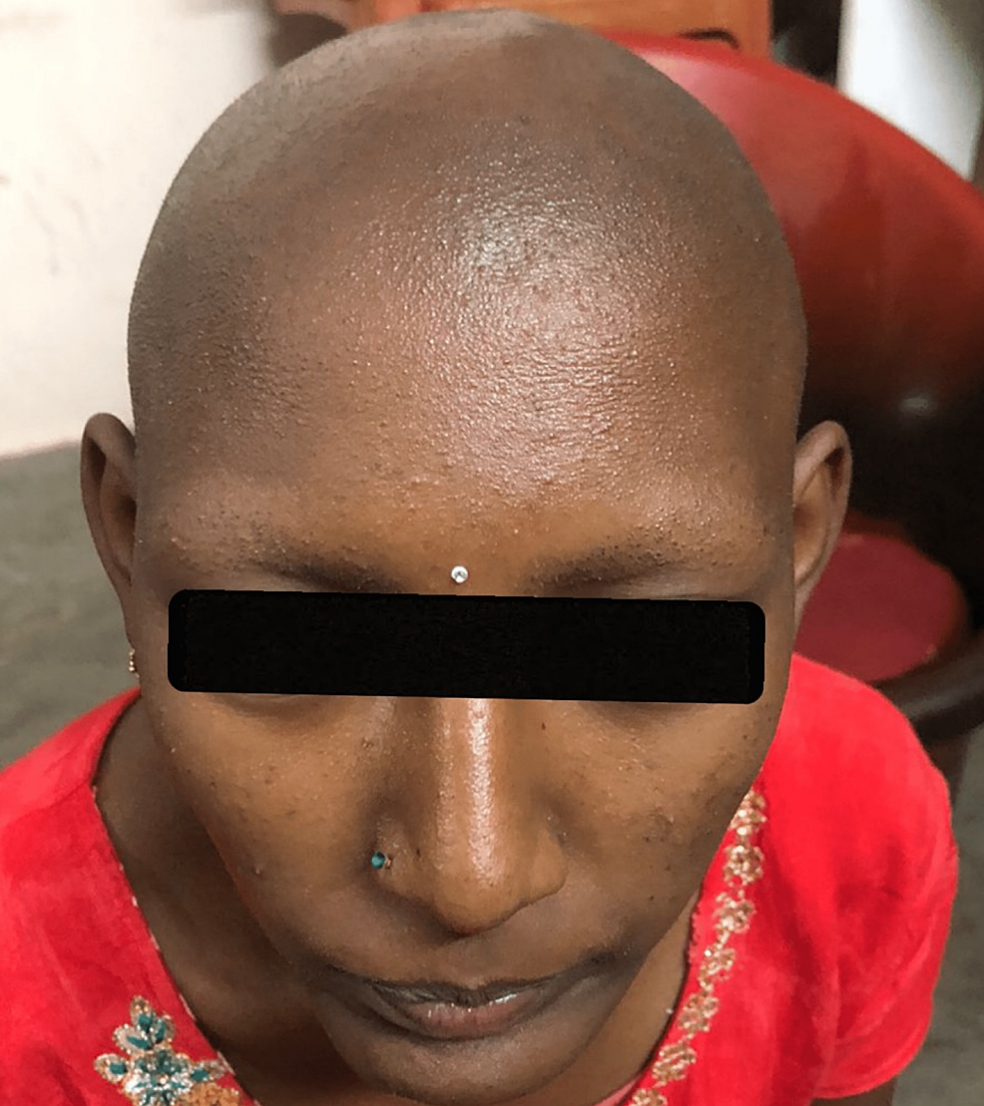
Complete atrichia of scalp, eyebrows and eyelashes

**Figure 5:**
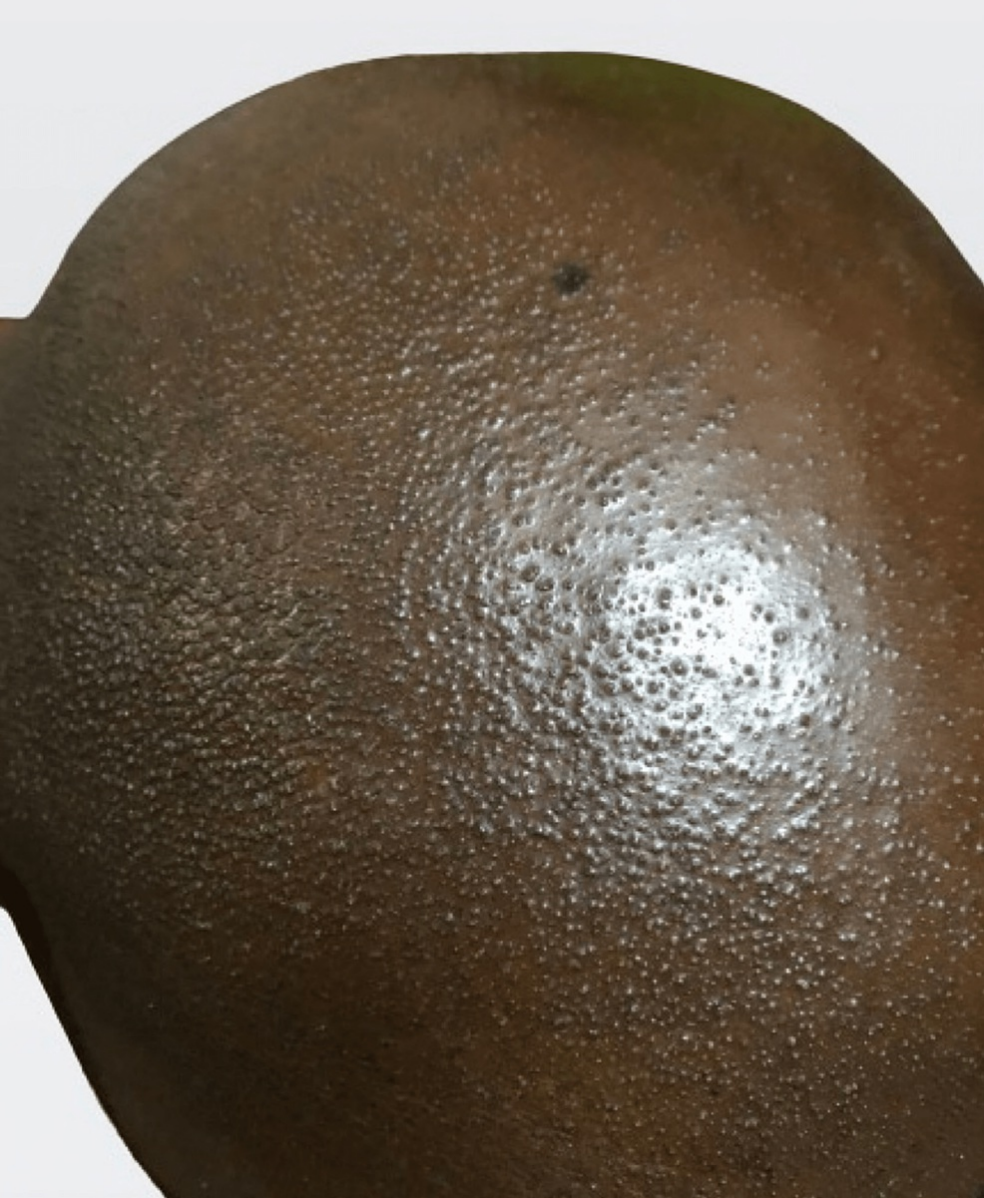
Papular skin lesions on the scalp

Here in this girl patient, there was no history of developmental and growth delays. There was no history suggestive of Vitamin D-dependent Rickets, such as bone deformities or bone aches, which can also present with baldness. There had been no previous history of hearing loss, seizures, or anhidrosis/hypohidrosis. Upon examination, all body hair including scalp hair, eyelashes, eyebrows, body hair, axillary hair, and pubic hair was completely absent (Figures 4, 5).

## Discussion

First criteria for diagnosing atrichia with papular lesions were established by Zlotogorski et al. [8] and later revised by Yip et al. [3], which had three out of five major criteria and three out of five auxiliary criteria. The case was classified as APL (Atrichia with Papular Lesions).

Congenital atrichia is an uncommon genetic disorder that can be inherited as an autosomal dominant trait or autosomal recessive or an X-linked pattern [9]. Familial cases of congenital atrichia are even rarer, as the mutated gene is usually not present in the family history. Damste and Prakken identified three unrelated women with a syndrome of alopecia and many follicular cysts that date back to early childhood as having APL, an uncommon illness [10], and it was Ahmad et al. who coined the term "congenital atrichia" following his observation of the hairless gene in various families around the world. Congenital atrichia with papular lesions, which is brought on by a mutation in the human hairless gene located on the short arm of chromosome 8, is one of the uncommon reasons of hair loss [11]. This condition has been also found to be relatively more popular among an ethnic minority known as Irish Travellers from Ireland. The relative incidence of uncommon gene abnormalities in this group has been attributed to a society with a consanguineous marriages and high fertility rates [12]. The hairless gene in this population has a recurrent splicing mutation that was previously identified in a family of English ancestry [13].

Atrichia with papular lesions is consistent clinically with hair loss over the scalp, sparse eyebrows and eyelashes, lack of secondary hair, formation of keratin-filled cysts and whitish hypopigmented streak over the scalp [11]. However, there is no irregularity of nails, teeth and sweating to a certain extent. On skin histology, hair follicles can either be completely absent from the skin, or they might be present in small, sporadic numbers.

Vitamin D-dependent rickets type IIA, hidrotic ectodermal dysplasia, Moynahan syndrome, and IFAP (Ichthyosis follicularis with alopecia and photophobia) syndrome have all been linked to it [13] and some rare associations with episcleritis [14], situs inversus and mesocardia [15]. According to a study by John et al. in two consanguineous Pakistani families, atrichia does indeed have a greater impact on these households. They discovered two new deletion mutations in exons 2 and 8 of the human hairless gene that result in frameshifts and downstream premature termination codons [16]. In this specific case, a marriage between third-degree relatives was identified. This case report describes the occurrence of familial congenital atrichia in a 16-year-old girl who acquired it from her parents. The clinical features of congenital atrichia are well-known, and our case report was consistent with the usual presentation of this condition. Patients with congenital atrichia are born with no hair on their scalp, eyebrows, and eyelashes, and they do not develop hair later in life.

Congenital atrichia must be diagnosed through genetic testing. Congenital atrichia is a condition where hair follicles are absent due to abnormalities in the HR gene, which encodes a transcription factor required for hair follicle development. As a result of the patient and her mother's lack of readiness for additional testing or therapy, we couldn't carry out a histological analysis or genetic testing nor provide her treatment for the same. Consequently, our scenario only covers three of the five main criteria as proposed by Yip et al. [3].

An individual's hair may reveal a lot of things about them [17]. Our patient's insecurity may stem not just from the hair loss, but from the standards society has set for an ideal human being [18]. The impact of permanent baldness and a hairless body can have both good and bad repercussions on her identity, depending on the individual's upbringing and social environment. Treatment of this condition has been found to be unresponsive to steroid therapy [17]. Given the lack of any really effective treatments for this ailment and the young age of the kid in question, we decided to focus on the psychological and social aspects of caring for those who suffer from it.

Patients with atrichia can benefit from support in a number of different ways, including by connecting with other people who may offer peer support and encouraging them to shift focus onto their own strengths and abilities rather than their physical appearance through open communication where patients with this condition can openly communicate about their feelings and struggles without fear of judgement. Alongside these steps, management of physical symptoms by wearing hats or wigs, and alternative learning choices can be explored like online education or homeschooling should be pursued if the child is having difficulty going to school. Hair prosthesis is another therapeutic option, the notion of which was first introduced in 1997 by a non-profit organization named 'Locks of Love' [19]. Despite the fact that our case did not permit any additional investigations or management, which could be due to a number of factors such as psychological, socioeconomical and lack of education, it is essential to remember that every person's experience with atrichia is different, and to treat each patient as an individual rather than a rare case. Thus it is prudent to effectively collaborate with the patient to determine the most effective strategies for the patient with congenital atrichia so as to suit his or her needs.

## Conclusions

Atrichia congenita with papular lesions is a rare, autosomal recessive form of total alopecia of the scalp, eyebrows, eyelashes, axillary, and pubic hair, characterised by hair loss shortly after birth and the development of keratin-filled cysts or horny papules over extensive areas of the body, including the face, neck, limbs, and trunk. The condition significantly affects the patient's confidence and way of life. It is probable that congenital atrichia with papular lesions is more prevalent than previously believed and that it is frequently confused with the putative autoimmune form of alopecia universalis. We anticipate that a greater awareness of this disorder will lead to a more accurate diagnosis and spare patients’ unnecessary treatment.
